# Drug Repurposing: A Review of Old and New Antibiotics for the Treatment of Malaria: Identifying Antibiotics with a Fast Onset of Antiplasmodial Action

**DOI:** 10.3390/molecules26082304

**Published:** 2021-04-15

**Authors:** Lais Pessanha de Carvalho, Andrea Kreidenweiss, Jana Held

**Affiliations:** 1 Institute of Tropical Medicine, University of Tuebingen, 72074 Tuebingen, Germany; lais_pessanha@hotmail.com (L.P.d.C.); andrea.kreidenweiss@uni-tuebingen.de (A.K.); 2 Centre de Recherches Medicales de Lambaréné (CERMEL), Lambaréné BP 242, Gabon

**Keywords:** antibiotics, drug repurposing, malaria, Plasmodium, slow and fast-acting drugs

## Abstract

Malaria is one of the most life-threatening infectious diseases and constitutes a major health problem, especially in Africa. Although artemisinin combination therapies remain efficacious to treat malaria, the emergence of resistant parasites emphasizes the urgent need of new alternative chemotherapies. One strategy is the repurposing of existing drugs. Herein, we reviewed the antimalarial effects of marketed antibiotics, and described in detail the fast-acting antibiotics that showed activity in nanomolar concentrations. Antibiotics have been used for prophylaxis and treatment of malaria for many years and are of particular interest because they might exert a different mode of action than current antimalarials, and can be used simultaneously to treat concomitant bacterial infections.

## 1. Introduction

Malaria is a vector-borne disease caused by protozoan parasites of the genus *Plasmodium* and is a major public health problem, mainly in Sub-Saharan Africa. Six *Plasmodium* species can cause malaria in humans, but only two species are clinically relevant. The two most virulent species are *Plasmodium falciparum* (most prevalent in Africa) and *P. vivax* (most prevalent in Southeast Asia and South America). The other species, namely *P. malariae, P. ovale wallikeri, P. ovale curtisi,* and *P. knowlesi* cause only a comparable negligible burden of disease. According to the latest World Health Organization (WHO) World Malaria Report, there were 229 million cases of malaria with 407,000 deaths, of which 67% were in children under five years of age [1].

*Plasmodium* parasites have a complex life cycle, including morphologically distinct forms in the vertebrate and mosquito hosts [2]. They require different times to complete their blood stage life cycle (24, 48, or 72 h, depending on the species) and the asexual blood stage is responsible for the clinical symptoms of the disease [3]. *P. vivax* and *P. ovale* can form dormant hypnozoites in the liver that can be reactivated weeks or years after the primary infection [4]. The pathogenesis of *P. falciparum* malaria is caused by the ability of asexual blood-stage parasites to cytoadhere to endothelial cells in the microvasculature of several organs, eventually causing severe symptoms and death [5]. In this context, the rapid onset of drug action to reduce parasite load is crucial to prevent the progression of uncomplicated malaria to severe disease or death.

### 1.1. Chemoprevention and Chemotherapy of Malaria

Due to increased efforts to eliminate malaria, the number of cases and deaths dropped from 2010 to 2016, but have remained stagnant since 2016 [1]. In the absence of an effective vaccine to prevent the disease, vector control, prophylaxis, and treatment by chemotherapies are the key options to combat it. First-line treatments for acute uncomplicated malaria are artemisinin-based combination therapies (ACT) that are highly efficacious to control the disease in endemic countries [1], while injectable artesunate (for at least 24 h) followed by a complete 3-day course of an ACT is recommended for severe malaria. For children under the age of 6 years with suspected severe malaria living in remote conditions, an initial treatment with artesunate suppositories is recommended to bridge the time until parenteral treatment can be initiated [6,7]. Clindamycin in combination with quinine is recommended to treat pregnant women during the first trimester, or in combination with artesunate or quinine for follow-up treatment of severe malaria when the mainstay ACT is not available [8]. Primaquine and tafenoquine are the only two approved drugs to eliminate hypnozoites, but only primaquine is used clinically to block transmission of *P. falciparum* and acts against stage V gametocytes [9].

The historic spread of anti-malarial drug resistance and the recent appearance of parasites with a delayed clearance phenotype following artemisinin intake, and ACT treatment failures in Southeast Asia emphasize the importance of the development of new alternative chemotherapies [10]. In addition, the use of primaquine and tafenoquine is restricted due to the potential of causing severe side effects in patients with glucose-6-phosphate dehydrogenase deficiency, a genetic human disorder present in malaria-endemic areas [11,12].

Currently, WHO recommends intermittent chemoprevention with sulfadoxine–pyrimethamine (SP) in sub-Saharan Africa, for certain groups, such as pregnant women and infants, and SP plus amodiaquine as seasonal malaria chemoprevention for children aged 3–59 months. However, the emergence of SP-resistant parasites, especially in east and southern Africa, jeopardizes the effectiveness of intermittent chemoprevention [13]. Doxycycline or atovaquone–proguanil are recommended for travelers to malaria-endemic areas. In addition, mefloquine, primaquine, and, since its approval in 2018, tafenoquine, have been recommended for chemoprophylaxis in travelers, depending on the circumstances by the Centers for Disease Control and Prevention (CDC) [14].

Despite the number of available antimalarial drugs, history has taught us that resistant parasites will arise and spread if drugs are in extensive use, threatening the lives of millions of people who are infected each year [15]. 

One strategy to quickly and cost-effectively find new treatment alternatives is to repurpose drugs approved for the treatment of other diseases [15].

### 1.2. Drug Repurposing

Drug repurposing, also referred to as drug repositioning, re-profiling, redirecting, etc., is the process of finding new medical uses for existing drugs. This is a very successful strategy, and around 25% of the annual income of the pharmaceutical industry comes from repurposed drugs [16]. The selection of drugs for the repurposing process can consider substances that were efficacious or not efficacious against the intended target disease in the clinical development program, as well as drugs removed from the markets due to unprofitability or other strategic reasons [17]. This approach has a lower risk of failure as the repurposed drug has already been shown to be safe for use in humans; it is also less time consuming as most pre-clinical tests have already been carried out successfully, also implicating that less investment is needed [15]. Such benefits are especially appealing for diseases where rapid discovery is needed (e.g., pandemics, such as coronavirus disease 2019 (COVID-19))and diseases with little financial investments in “de novo” drug discovery as neglected tropical diseases [18].

### 1.3. Antibiotics for the Treatment of Malaria

Antibiotics are substances with antibacterial properties that mainly target cell wall, nucleic acid, or protein synthesis [19]. Some antibiotics have been tested and used for a long time as alternative antimalarials, as reviewed previously [20,21]. For example, as mentioned above, the use of clindamycin for severe malaria, doxycycline for prophylaxis in travelers, and SPfor prophylaxis of risk groups in endemic regions. Active antibiotics for malaria treatment are of great interest and importance as coinfections/bacteremia can occur concomitantly during Plasmodia infections, being potentially a life-threatening factor mainly for sick children [22,23].

Unfortunately, many of the antibiotics have a slow onset of action against apicomplexan parasites, resulting in a so-called delayed death effect. This means that the antibiotics need two replicative cycles to exert their action against the parasites. This slow onset of action cannot only be seen in vitro, but also in vivo in monotherapy treatments of malaria, and should therefore not be considered as the main chemotherapy to treat acute *P. falciparum* malaria [24,25]. The reason for the delayed death phenotype has been partly elucidated recently. So far, all antibiotics that lead to a delayed death in Plasmodia target the housekeeping functions of the apicoplast, a relic plastid that arose by endosymbiosis of a cyanobacterium [26,27]. The apicoplast is found in apicomplexan protozoans and has housekeeping metabolic pathways, such as isoprenoid precursor, fatty acid, and heme biosynthesis, and Fe-S cluster assembly [28,29,30]. The type II fatty acid and heme biosynthesis were shown to be dispensable in the erythrocytic stage [31,32], the remaining isopentenyl pyrophosphate (IPP) production was the sole required function in the blood-stage *Plasmodium species* [33]. This hypothesis was proven by the in vitro supplementation of antibiotic-treated parasites with IPP, which could rescue the parasites from the toxic effect caused by the antibiotics and resulted in normally replicating apicoplast-free parasites [33]. Most antibiotics that target the apicoplast lead to the inheritance of a defective organelle only in the progeny parasites, which then fail to grow and subsequently die [34].

On the other hand, some antibiotics that disrupt essential apicoplast metabolic pathways are already highly active in the first replicative cycle [35]. Antibiotics directly targeting the isoprenoid synthesis in the apicoplast but not replication, transcription, or translation of the apicoplast genome, show a fast onset of action. In addition to the apicoplast, some antibiotics might have other/additional targets as activity can be seen experimentally against apicoplast-free parasites with IPP supplementation. This group of antibiotics deserve particular attention as they show a fast onset of action. In general, antibiotics are of special interest as concomitant bacterial and plasmodial infections are common in febrile African children that could lead to complications and more severe infections if untreated. Another advantage is that no resistances against antibiotics (besides folate inhibitors) of Plasmodia have been confirmed so far.

## 2. Methods

For this review, an extensive literature search was conducted to identify publications on the antimalarial activities of antibiotics that are commercially available or have already undergone clinical tests. The key studies are summarized in Table 1 and Table 2.

**Figure 1:**
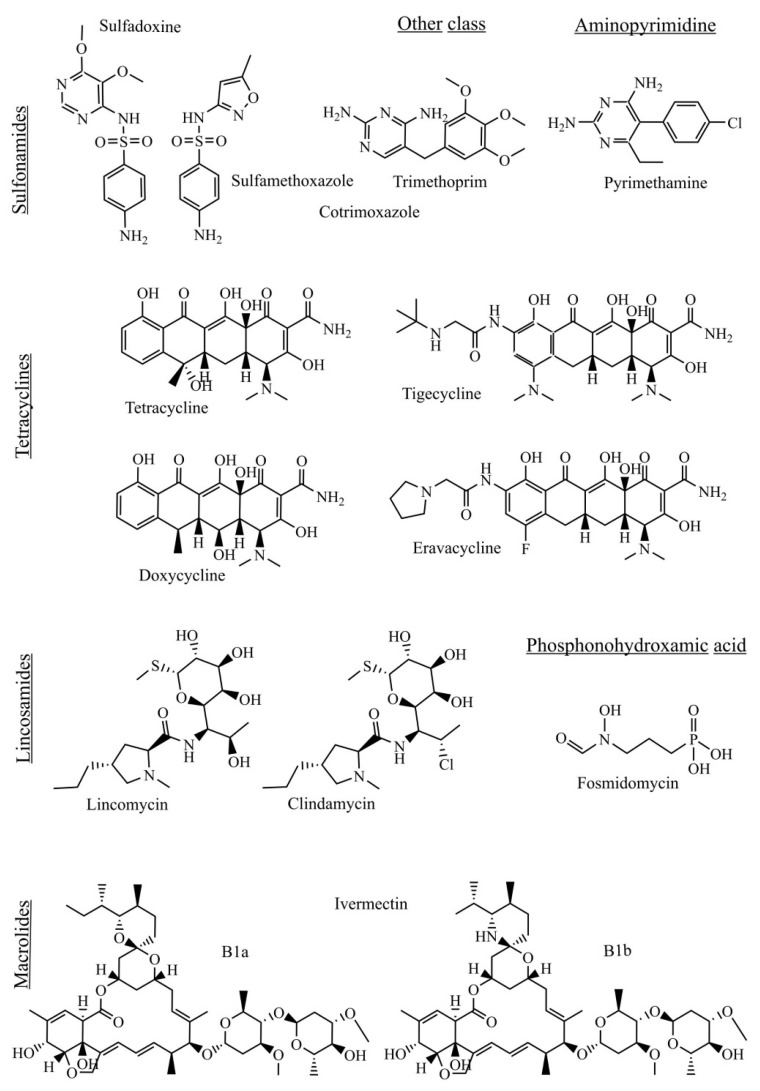
Antibiotics with potent antiplasmodial activity.

**Figure 2:**
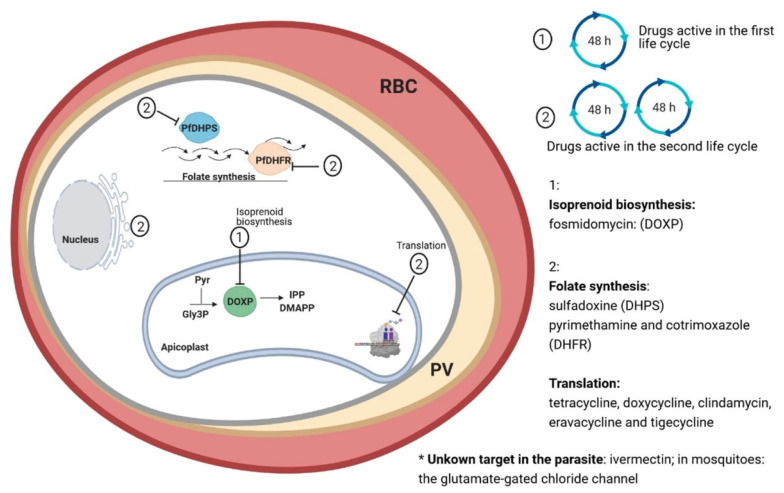
Targets of antibiotics with antiplasmodial activity.

We considered antibiotics to be all naturally or synthetically produced compounds with antibacterial activity. Historically, macrolides, tetracyclines, quinolones, and lincosamides have been the most promising classes of antibiotics for antiplasmodial activities, and recently tested antibiotics confirmed this prediction. Figure 1 and Figure 2 show the fast-acting antibiotics and their targets in Plasmodia. On the other hand, many classes of antibiotics, such as nitroimidazoles, cephalosporin, oxazolidinones, and amphenicols tested against *Plasmodium* species were completely inactive. Herein, we focused on antibiotics that showed an in vitro antiplasmodial activity in nanomolar concentrations to identify antibiotic compound classes that could display a fast onset of activity.

## 3. Fast-Acting Antibiotics

### 3.1. Folate Synthesis Inhibitors

After the emergence of widespread resistance by the malaria parasite in endemic regions to the widely used 4-aminoquinoline chloroquine, the folate synthesis inhibitor sulfadoxine-pyrimethamine (SP) was introduced as a first line treatment in the 1990s, as a cheap and efficacious alternative [36]. Sulfadoxine, as all sulfonamides and sulfones, is an analogue of *p-*aminobenzoic acid and inhibits dihydropteroate synthase (DHPS), a key enzyme in the biosynthesis of folate [37]. Pyrimethamine is a competitive inhibitor of dihydrofolate reductase (DHFR), a key enzyme in the redox cycle for the production of tetrahydrofolate [38,39]. Both enzymes are required for the biosynthesis of DNA and proteins, but point mutations in the dhps and dhfr domain led to resistant parasites [40,41,42]. As resistances emerged quickly, treatment recommendations changed again in 2006 to the today used ACTs as first line treatments [36]. SP, however, is still the only recommended drug for preventive treatment of malaria in pregnant women in endemic areas (IPTp: intermittent preventive treatment in pregnancy), and in infants (IPTi), and for seasonal malaria chemoprophylaxis in children under 5 years of age when combined with amodiaquine in regions with seasonal malaria [43].

Another fixed drug combination normally not used as an antimalarial targeting folate synthesis is cotrimoxazole, which consists of trimethoprim and sulfamethoxazole (1:5 ratio). Trimethoprim is a synthetic antibiotic that belongs to the diaminopyrimidine class and has a broad spectrum of activity on bacteria by inhibiting the enzyme dihydrofolate reductase. Trimethoprim was introduced in 1960s, and was frequently used in combination with sulfonamides to treat urinary tract infections [44]. Sulfamethoxazole is a sulfonamide drug and interferes with the synthesis of folate in bacteria by competing with p-aminobenzoic acid [45].

Daily cotrimoxazole prophylaxis of HIV-infected patients is a well-known strategy to avoid opportunistic infections [46], and several studies confirmed that this also reduced the risk of *Plasmodium* infections in malaria endemic areas [47,48,49]. A study conducted in 1971 showed the combination of trimethoprim and sulfamethoxazole to be efficacious to treat children aged 5–12 years with uncomplicated malaria in Nigeria at a concentration of 8 mg/kg of trimethoprim and 40 mg/kg of sulfamethoxazole [50]. Years later, in the year 2000, cotrimoxazole was still shown to be efficacious to treat children with uncomplicated malaria in highly-endemic areas of Kenya, Malawi, and Nigeria [51,52]. In addition, continued use of cotrimoxazole decreased parasite load and suppressed malaria symptoms [53]. A systematic review and meta-analysis investigating the role of cotrimoxazole prophylactic treatment in preventing malaria in children in sub-Saharan Africa confirmed the large impact of the intervention on malaria incidence and mortality [54].

Due to the extensive evidence of the potent activity of cotrimoxazole against malaria and its broad activity against many microorganisms, this drug was recommended by WHO in 2006 as a prophylactic treatment for HIV-infected children to prevent plasmodial and bacterial infections. In addition, cotrimoxazole was also indicated for HIV-exposed uninfected children from 6 weeks of age if breastfed (WHO 2016), and for persons living with HIV [51,55] since the HIV infection may suppress the immune response to malaria [56]. Despite the numerous advantages of cotrimoxazole, such as its low price and safety, its use is associated with major concerns, including the fear that it might favor the increase of antifolate resistance in *Plasmodium* parasites, leading to cross-resistance, for example, to SP. However, a recent study showed that cotrimoxazole can control malaria infections even in regions of a high prevalence of SP-resistant parasites, and there was no evidence that its use selects for mutations that confer SP resistance [57]. Cotrimoxazole was in development as antimalarial by the Institute of Tropical Medicine Antwerp, but development has not been progressing recently (Medicines for Malaria Venture - MMV Global Portfolio of Antimalarial Medicines) [58].

Currently, formulation improvements are ongoing, i.e., a new pediatric formulation of SP plus amodiaquine has been recently approved (SPAQ-CO™/Supyra^®^ (sulfadoxine-pyrimethamine + amodiaquine) for seasonal preventive treatment of malaria and further formulation improvements are in development, especially adapted to the pediatric population. In addition, new formulations of only SP are in the patient confirmatory phase (MMV Global Portfolio of Antimalarial Medicines) [58].

Therefore, overall, the already widespread use of folate synthesis inhibitors limits the potential of new/additional folate synthesis inhibitors for additional applications in malaria therapy.

### 3.2. Tetracyclines

The first tetracyclines, chlortetracycline and oxytetracycline, were discovered in the late 1940s as products of *Streptomyces aureofaciens* and *Streptomyces rimosus,* respectively. Tetracycline antibiotics consist of a linear fused tetracyclic core to which different functional groups are attached [59]. They can be classified into four groups, called first-generation (1948–1963), second-generation (1965–1972), glycylcyclines, and the new tetracyclines (eravacycline, sarecycline, and omadacycline) [60]. Tetracycline is a class of antibiotics that shows broad-spectrum activity against gram-positive and gram-negative bacteria, as well as protozoan parasites. In bacteria, the mode of action is related to the binding to the highly conserved 16S ribosomal RNA present at the bacterial 30S ribosomal subunit. Tetracyclines, including doxycycline and tigecycline, impair the translation by sterically arresting the docking of aminoacyl-transfer RNA during the elongation process [61,62]. A well-known side effect caused by tetracyclines is the discoloration of primary and permanent teeth caused by the chelation of calcium ions and absorption by tissues that are calcified during the treatment [63]. The use of tetracyclines to treat malaria dates back to the 1950s, when aureomycin, chlortetracycline, and oxytetracycline were used successfully to treat patients with uncomplicated *P. falciparum* and *P. vivax* malaria infections [64,65,66], while doxycycline has been used for prophylaxis since 1985 [67]. Tetracyclines usually suffer from the shortcoming of a slow onset of action. However, one novel tetracycline, tigecycline, was reported to be fast-acting in two studies [68,69], but this could not be confirmed by others [70].

Tigecycline, the first marketed glycylcycline is a semisynthetic 9-*t*-butylglycylamido derivative of minocycline especially designed to overcome the mechanisms of tetracycline resistance in bacteria [71]. Its antiplasmodial activity was first evaluated in clinical isolates of *P. falciparum* from Bangladesh [68], and the short (one parasite cycle) assays showed an half maximal inhibitory concentration (IC50) value of 699 nM. Another study was performed with clinical isolates of *P. falciparum* from the Brazilian Amazon and showed similar results with an IC50 of around 600 nM in an assay evaluating only activity after the first cycle [69]. In vivo activity of tigecycline was evaluated in *P. berghei* infected mice at concentrations of 3.7, 11.1, 33.3, and 100 mg/kg/day given for four days [72]. Although already the concentration of 3.7 mg/kg/day reduced the parasitemia by 77% and 91% on day 5, only the regimens with 100 mg/kg/day cured the mice completely when assessed on day 28. A third study assessed the in vitro activity of tigecycline in culture-adapted strains and clinical isolates from Gabon [70], showing a delayed death effect as IC50s after 6 days (at least two parasite cycles) of incubation (~ 200 nM) were tenfold lower than after 3 days (~ 2.5 µM). Similar results were also observed for the clinical isolates. Another novel tetracycline, eravacycline, also showed improved activity compared to other tetracyclines in vitro against *P. falciparum* when analyzed over two cycles (IC50 of 14 nM) [73]. Even though the delayed death effect was seen in laboratory strains, this was not confirmed in clinical isolates, where the compound was already active in the short assay (three days). Shortcomings of these novel tetracyclines is that they can only be given parenterally.

Overall tetracyclines have proven to be valuable drugs for antimalarial therapy—novel tetracyclines might even show improved and accelerated activities and should be screened for their suitability to offer improved antimalarials.

### 3.3. Fosmidomycin

Fosmidomycin is a hidroxylaminopropylphosphonic acid isolated from *Streptomyces lavendulae* in 1979 [74]. Fosmidomycin (originally called FR-31564) and another unsaturated product of *S. lavendulae* (FR-32863) were highly active against Gram-positive and Gram-negative bacteria, including clinical isolates of multidrug-resistant *Escherichia coli, Klebsiella pneumoniae, Enterobacter cloacaea, Pseudomonas aeruginosa,* and *Mycobacterium tuberculosis* [75,76]. Of the two compounds, only fosmidomycin showed promising antiplasmodial activity.

Fosmidomycin inhibits the 1-deoxy-*D*-xylulose 5-phosphate reductoisomerase (DOXP), a key enzyme of the methylerythritol phosphate pathway (also called non-mevalonate pathway) (MEP) [77]. The first evidence on its mode of action came from its antimicrobial spectrum of activity, as only the group of microorganisms possessing the DOXP enzyme was affected by the compound. The MEP is found in Gram-negative bacteria, some Gram-positive bacteria, plastid-containing eukaryotes and plants, whereas humans and mammals possess the homologous mevalonate pathway. Both pathways are responsible for the production of the isopentenyl pyrophosphate (IPP) and its isomer dimethyl allyl pyrophosphate, which are isoprenoid precursors [78]. The discovery of MEP in Plasmodium parasites for IPP biosynthesis suggested fosmidomycin as a possible antimalarial. Supplementation with IPP in vitro can overcome the activity of fosmidomycin. This direct inhibition of the pathway in the apicoplast by fosmidomycin leads to a fast, first cycle growth inhibition. In fact, fosmidomycin could impair the growth of different strains of *P. falciparum* in vitro in submicromolar concentrations after one replicative cycle [79] and cured *P. vinckei-*infected mice when administered every 8 h for 4 days [79]. This is one of the few drugs that target the apicoplast, but does not display the delayed death effect. Shortcomings of fosmidomycin include its moderate bioavailability (10–30%) and its relatively short half-life (1.9 h in plasma) [80].

A clinical trial performed in Gabon and Thailand assessed the efficacy and safety of fosmidomycin monotherapy to treat adults with uncomplicated falciparum malaria with an oral dose of 1200 mg every 8 h for 7 days [81]. All patients were cured on day 7, but recrudescent parasitemia was found in 2/9 patients from Gabon and 7/9 patients from Thailand at the end of the follow-up on day 28. To improve the dose regimen, a subsequent study was performed in Gabon to evaluate the same dosage but in a shorter regimen of 3–5 days, concluding that the minimum time of fosmidomycin treatment to cure malaria was 4 days [82]. The antibiotic clindamycin was proposed as a good partner drug after demonstration of synergistic activity in vitro as well as in a mouse model [83]. Clindamycin shows a delayed death phenotype and has a half-life of 2–3 h [32]. The efficacy of the combination for the treatment of uncomplicated malaria was shown in adults and older children in different treatment regimens [84,85,86,87], but the selected 3-day regimen of fosmidomycin and clindamycin of 30 and 10 mg/kg of body weight, respectively, every 12 h, was not efficacious enough to cure malaria in children younger than 3 years [87,88]. Fosmidomycin was subsequently evaluated together with artesunate in five different regimens of 1–5 days, showing that a 2-day regimen is sufficient to achieve cure of malaria in 10/10 children (6–12 years) on day 14, and a 3-day regimen (or longer) in 10/10 children (6–12 years) on day 28 [89].

Fosmidomycin was proposed to be developed, together with piperaquine, by Deutsche Malaria GmbH (DMG), according to the Global Portfolio of Antimalarial Medicines [58]. In addition, a triple combination of artesunate, clindamycin, and fosmidomycin is in development (Pan African Clinical Trials registry: PACTR202008909968293).

### 3.4. Macrolides

Macrolides are compounds that can contain one or more lactone rings with 8 to 62 atoms [90]. The most common ones have a lactone ring containing 14, 15, or 16 atoms (erythromycin-like, azithromycin-like, and josamycin-like, respectively). Macrolides can consist of simple or complex lactones containing amino nitrogen, amide nitrogen, a thiazole ring, or oxazole ring in their skeletons [91]. In bacteria, macrolides act as inhibitors of the protein synthesis. In detail, the activity is related to the impairment of the passage of new polypeptides over the nascent peptide exit tunnel of the bacterial ribosome during protein translation [92].

Depending on the time needed to exert their effects against Plasmodia, macrolides can be grouped as either slow or fast-acting drugs. In the first group are erythromycin and its semisynthetic derivatives, while in the second group are other antibiotics such as avermectins, borrelidin, and kitasamycin (also called leucomycin). While antibiotics from the first group were poorly active against Plasmodia, some antibiotics from the second group showed a fast onset of activity. Borrelidin and kitasamycin for example, showed an IC50 in the low nanomolar range (2 and 50 nM) when tested in vitro. In vivo tests in the *P. yoeli* murine malaria model confirmed their potent antiplasmodial activity [93,94]. However, these two drugs will not be discussed here in detail because no clinical data are available.

Currently, a promising compound for preventing transmission of malaria parasites is ivermectin. It is not an antibiotic in the classical sense but an antiprotozoal macrolide drug derived from avermectin B. Avermectins are 16-membered macrocyclic lactones naturally produced by *Streptomyces avermitilis*. Four homolog pairs containing a major and minor component of avermectins were isolated. Avermectin B and its derivatives (avermectin B1a and avermectin B1b) are the most active macrolides against endo- and ectoparasites [95]. Ivermectin, the most studied semi-synthetic derivative, is a mixture of avermectin B1a (> 80%) and B1b (< 20%) showing a broad-spectrum activity against different parasitic diseases such as human onchocerciasis, strongyloidiasis, ascariasis, trichuriasis, lymphatic filariasis, scabies, and enterobiasis [96,97,98,99,100]. In addition to the anti endo- and ectoparasite activities, ivermectin was also reported to be active against multidrug-resistant strains of *Mycobacterium tuberculosis* in vitro [101].

The potent effect of ivermectin on nematodes is related to its selective and high-affinity binding to different kinds of chloride channels in invertebrate muscle and nerve cells. Consequently, the cell membrane permeability increases, and the chloride ions cause hyperpolarization of the cells, leading to paralysis and parasite death [102]. In Plasmodia, a different mode of action is proposed, which might be related to the inhibition of the nuclear import of signal recognition particle polypeptides, impairing parasite growth; however, this is not yet completely elucidated [103]. Ivermectin has been licensed for human use more than 30 years ago, and more than 2.7 billion 150–200 µg/kg single doses have been distributed in the African region through the Mectizan Donation program, mainly to fight onchocerciasis [104], and was shown to be generally safe outside *Loa loa* endemic areas, with only rarely occurring adverse events [105,106]. However, in individuals with a high *L. loa* microfilarial load, ivermectin can cause severe encephalopathy [107]. In onchocerciasis-infected patients, adverse events to ivermectin are associated to the intensity of microfilarial infection and primarily characterized as mild and transient reactions due to dying microfilaria (called Mazzotti type reactions), usually waning with subsequent administrations [106,108]. Ivermectin is not recommended for use in pregnant women, children under the age of five years, and in areas co-endemic for *L. loa* [109].

In the last years, ivermectin has been extensively investigated as a potential tool to control the transmission of malaria parasites [110,111,112]. The fact that ivermectin has a wide spectrum activity as endectocide, could induce a reduction in malaria transmission by causing death of the mosquitoes feeding on the treated population. As shown in different studies, when an *Anopheles spp.* mosquito took a blood meal from a recently treated host, its survival was reduced [113,114] and *Plasmodium* sporogony was impaired [115,116,117]. Repeated ivermectin mass drug administration during the malaria transmission season reduced the incidence of malaria episodes in children in a trial in Burkina Faso (mean 2.0 episodes per child in the treated group, versus 2.5 in the untreated) [118]. Ivermectin was also evaluated together with standard antimalarial treatments to reduce post-treatment Plasmodia transmission [119].

Ivermectin could have additionally an activity on its own against human *Plasmodium* stages. Evaluations showed in vitro activities of ivermectin against asexual *P. falciparum* stages (IC50 of around 100 nM), and gametocytes, even though only at quite high concentrations (IC50 ~ 500 nM) [120]. In addition, ivermectin impaired the infection of human hepatoma cells by *P. berghei* in vitro (IC50 of around 2 µM) [121] and in vivo reduced blood-stage parasitemia (~ 80% after all dosages) in *P. berghei* infected mice after three-doses of 1–10 mg/kg [121]. However, the recommended dose for standard treatment in humans does not reach the needed plasma levels to show an effect. As demonstrated, a single dose of ivermectin at 0.4 mg/kg, 2 h before volunteers were experimentally infected intravenously with *P. falciparum* sporozoites did not prevent further infection [122]. Other studies showed that also higher doses of ivermectin were well tolerated in adults with multiple doses up to 3 days of 600 µg/kg per day or up to a single dose of 2000 µg/kg [123,124,125,126]. Whether higher doses are safe and whether additional benefits justify their administration is currently being investigated. For transmission control the development of derivatives with a longer half-life (~ 38 h after a single dose of 12 mg ivermectin) [127], might be of value.

Recently, the antimalarial activity of the first ivermectin hybrids (with triazoles, ferrocene-based and dihydropyrimidine derivatives) was assessed and showed that the most active derivative was threefold and tenfold more active than ivermectin against hepatic and blood-stage infections, respectively [128]. Currently, further clinical trials are ongoing to investigate the potential of ivermectin to control and block parasite transmission, the safety of higher doses, and the treatment of asymptomatic infected patients (see clinical trial.gov NCT03967054 and the Pan African Clinical Trials Registry pactr.samrc.ac.za PACTR201907479787308).

## 4. Lincosamides

Lincosamides constitute a group of antibiotics derived from the natural products lincomycin and celesticetin, which are produced by many *Streptomycin spp.* [129]. Lincosamides comprise a substituted proline moiety and an unusual thiooctose sugar connected via an amide bond [130]. Celesticetin is less effective than lincomycin in vivo and in vitro [129]. The antimicrobial effect of lincosamides is related to the inhibition of the formation of proteins by binding to the 50S subunit on the ribosome, impairing the docking of charged tRNAs and their movement through the peptidyl transferase center [131].

The structure of lincomycin consists of a trans-*N*-methyl-4-*n*-*L*-proline (propylhygric acid) linked by a peptide bond with the sugar 6-amino-6,8-dideoxy-1-thio-*D*-erythro-α-*D*-galactopyranoside (methylthio–lincosamide) [132]. Replacement of hydroxyl in the side chain by chlorine produces clindamycin, the most used lincosamide drug in clinical practice [129]. Clindamycin monotherapy was first reported to treat malaria successfully in 1975 [133], thereafter many clinical trials were performed to assess the efficacy of clindamycin monotherapy or in combination with other drugs to treat malaria, as reviewed in [134]. Clindamycin monotherapy was administered in more than 500 semi-immune and nonimmune patients in South America, Africa, and Southeast Asia. As concluded in this review, clindamycin monotherapy had an efficacy of 98% when given for at least 5 days and at least twice daily, with a mean parasite clearance times between 4 and 6 days and fever clearance times in the range of 3 to 5 days. Clinical trials with clindamycin–quinine combinations to treat children, pregnant women, and both semi-immune and nonimmune adults, showed an efficient alternative with a reduction in the treatment time from at least 7 and 5 days when quinine and clindamycin are administered alone, respectively, to 3 days in combination. In addition, clindamycin–chloroquine combination was administered in children and semi-immune adults in Gabon, where the rate of chloroquine resistance is high. The cure rate of the combination therapy in children was 70% (dosage of 5 mg/kg of clindamycin, twice daily for 3 days, plus a total dose of 25 mg/kg of chloroquine) and 94% (5 mg/kg of clindamycin, twice daily for 3 days, plus a total dose of 45 mg/kg of chloroquine), in comparison to 9% and 32% when chloroquine was administered alone, respectively.

Clindamycin monotherapy cannot be recommended due to the slow onset of action, but the combination of clindamycin with quinine is recommended by WHO to treat pregnant women during the first trimester, or in combination with artesunate or quinine when an ACT is not available [8]. Lincosamides are very active antimalarials with very low nanomolar IC50s in vitro [134], but we do not consider them further here, as we could not identify compounds with a fast onset of action.

## 5. Conclusions and Perspectives

Due to the ability of *P. falciparum* to develop resistances to nearly all drugs in widespread clinical use and the risk of spreading of these resistant parasites, new alternatives to treat the disease are constantly needed. In particular, novel compounds for the development of non-ACT combinations are urgently needed. In this context, drug repurposing is particularly attractive tool for discovery of new treatments for malaria and other neglected tropical diseases as costs of clinical development are reduced for this inherently resource limited market.

Among the advantages to use antibiotics already approved, one can list the reduced costs of clinical development, their worldwide availability, and the possibility to treat different infections at the same time. Many antibiotics target the plasmodial apicoplast, an organelle that is not present in human cells and, thus, a highly selective drug activity can be expected. Furthermore, the apicoplast is a target different from common antimalarials in use and, therefore, cross-resistance should not be found. Several antibiotics have already been used/are in use for prophylaxis, or as alternative partner drugs for the treatment of malaria, and were shown to be valuable, but others deserve to be investigated further. In particular, the development of compounds with novel chemical modifications should be closely observed. As listed in this review, there are several antibiotic classes that show promising activities, with some being already highly active from the first cycle of parasite replication.

The main target of many antibiotics (not ivermectin and folate synthesis inhibitors) in Plasmodia is a single organelle, the apicoplast. Even though the main target organelle is the same, some of them show a fast onset of action and others are only active starting from the second cycle. According to current knowledge, slow-acting antibiotics impair the import and processing of nuclear-encoded proteins to the apicoplast or apicoplast DNA replication, compromising the essential metabolic pathways of the organelle, but only for the progeny. On the other hand, studies on fast-acting antibiotics suggest that the direct chemical or genetic disruption of metabolic pathways causes immediate parasite death, as in the case of fosmidomycin [33].

Although the use of antibiotics as antimalarial drugs seems to be a promising alternative, it must be considered that the uncontrolled use of antibiotics could also promote the emergence of resistant bacteria. This has been investigated for the prophylactic use of doxycycline against malaria in military personnel [135,136] and has been discussed previously [19]. Therefore, the pros and cons of the use of antibiotics for malaria treatment should be carefully weighted and eventually restricted to certain groups of patients [137].

## Figures and Tables

**Table 1:** Summary of the pre-clinical data of fast-acting antibiotics.

Antibiotic	Outcomes – Pre-Clinical Data			
	In vitro (IC50)	Ref	In mice	Ref
**Tigecycline**	One-cycle assay: 66 clinical isolates from Bangladesh with parasite density of 8311–13,735/µL: mean 699 nM (range: 496–986).	[68]	*P. berghei* infected mice (5 per group) were treated with 3.7, 11.1, 33.3 and 100 mg/kg for 4 days. Only the treatment with 100 mg/kg/day cured the mice on day 28.	[72]
	One-cycle assay: DW2: 568; 3D7: 332; 3 clinical isolates from Brazil: mean ~ 600 nM (range 344–726).	[69]		
	One-cycle assay: IC50 3D7: 2300 nM; Dd2: 2800 nM; Two-cycle assay: IC50: 3D7: 220 nM; Dd2: 173 nM;	[70]		
	Two-cycle assay: 23 clinical isolates from Gabon with a mean parasite density of 45,174/µL (range: 750–93,827): IC50: 160 nM (range: 114–223).			
**Eravacycline**	One-cycle assay: 3D7: IC50: 1996 nM; two-cycle assay: 14 nM. Thirty-three clinical isolates from Gabon with parasitemia at 0.05%.	[73]		
	One-day assay: IC50: 69 nM (range: 35–142); two-day assay: IC50: 29 nM (range: 13–157).			
**Fosmidomycin**	One-cycle assay: IC50 3D7:150 (range:100–240); HB3:71 (range: 46–140); Dd2:170 (range: 120–260); A2:150 (70–260);	[83]	Five *P. vinckei* infected mice were treated with 30 mg/kg daily for 8 days. All mice were cured on day 28.	[79]
	One-cycle assay with 3D7: FMD showed synergism with CLD (FIC: 0.43); additive effect with doxycycline (FIC: 0.93), quinine (FIC: 0.93) and azithromycin (FIC: 0.84).		*P. vinckei* infected mice were treated for 2 days with 75 mg/kg FMD and 5 mg/kg CLD separately or in combination. The parasitemia of mice treated with FMD or CLD was 7.8 and 20% on day 3, respectively, while the controls had 42%. The combination reduced the parasitemia to 0.1% on day 3 and 0.2% on day 5.	[83]
**Ivermectin**	One-cycle assay: IC50 (mean) 3D7: 100 nM; Dd2: 110 nM; K1:365 nM; clinical isolates from Gabon (0.05% parasitemia): IC50 (mean) ~ 100 nM mature gametocytes: 500 nM.	[120]	Three × 10 mg/kg reduced ~ 80% of P. berghei load in mice 46 h after infection.	[121]
	In vitro addition of 2 µM IVM impaired human hepatoma cells infection by P. berghei	[121]		

FMD: fosmidomycin; CLD: clindamycin; IVM: ivermectin; FIC: fractional inhibitory concentration.

**Table 2:** Summary of the clinical data of fast-acting antibiotics to treat malaria in humans.

Clinical Data	Ref
**Cotrimoxazole**	
Three-hundred HIV-infected Ugandan children received CTM prophylaxis, while 561 healthy children	[47]
were followed as control. After 11 months, only nine cases of malaria were diagnosed	
among children taking CTM prophylaxis, in comparison with 440 children in the control group.	
HIV-uninfected Ugandan children aged 6 weeks to 9 months breastfed on HIV-infected	[48]
mothers received CTM syrup (40 mg TM and 200 mg SFM) at the following doses:	
2.5 mL/day for children ≤ 4 kg, 5 mL/day for children > 4–8 kg, and 10 mL/day for children	
> 8–15 kg. Children weighing 10–15 kg received CTM tablets (80 mg TM and 400 mg SFM)	
and were prescribed one tablet daily thereafter. After cessation of breastfeeding,	
HIV uninfected children were randomized to CTM prophylaxis (n = 87)	
or to continue daily CTM prophylaxis until 2 years of age (n = 98); 699 episodes of malaria	
in total: 299 episodes in the prophylaxis group and 400 episodes in the discontinued group.	
HIV-infected patients aged 18 years or older received CTM daily prophylaxis in Uganda.	[49]
Baseline incidence of malaria was 50 episodes per 100 person-years during a 154-day follow up	
(466 participants). CTM prophylaxis was associated with 9 episodes of malaria per 100 person-years	
during 532-day follow-up (399 participants) (76% lower malaria rate), and CTM + ART	
was associated with 3.5 episodes per 100 person-years during a 126-day follow-up	
(1035 participants) (92% lower malaria rate).	
In Nigeria, a single dose of 8 mg/kg of TM + 40 mg/kg SFM cured all 42 children aged 5–12 years	[50]
with UFM on day 3. On day 14, all patients were still negative, but on day 67, 24 out of 36 patients were positive again.	
A total of 102 Nigerian children aged 0.5–12 years with UFM were treated with 20 mg/kg	[51]
CTM, twice daily for 5 days: on day 7, they had lower propensity to develop gametocytes	
than SP (34 versus 63%), checked by light microscopy.	
In Malawi, 205 children aged 0.5–5 years with UFM received CTM or SP for 5 days plus	[52]
ERY 125 mg 4 × day < 10 kg; 250 mg > 10 kg. Eighty-seven percent of children receiving CTM and 80%	
receiving SP reached adequate clinical responses on day 14. On day 7, gametocyte	
prevalence was 55% and 64% among children receiving CTM and SP, respectively.	
In Kenya, 500 participants ≥ 18 years old, HIV-positive, and taking first-line AS and	[57]
CTM were randomized to discontinue with CTM prophylaxis (STOP-CTM; 250 individuals)	
or continue (CTX; 250 individuals). Blood samples were collected at months 0, 3, 6,	
9 and 12. The prevalence of mutant haplotypes associated with SP-resistant parasites	
in pfdhfr (51I/59R/108N) was 52% in the STOP-CTM arm versus 6.3% in the CTM arm.	
The pfdhps (437G/540E) was found in 57% in the STOP-CTM and 25% in the CTM arm.	
**Fosmidomycin**	
A total of 11 Gabonese and 15 Thai adults with UFM were treated with 1200 mg every 8 h for 7 days.	[81]
Seventy-eight percent of Gabonese and 22% of Thai patients were cured on day 28.	
A total of 27 Gabonese adults with UFM: 1–2 g every 8 h for 3, 4, or 5 days,	[82]
cure rates on day 14: 60, 88 and 89%, respectively.	
In Thailand, 70 patients with 15–61 years old with UFM were treated with two regimens of FMD in combination	[84]
with CLD. Group I: FMD (900 mg) and CLD (300 mg) every 6 h for 3 days	
(n = 25); Group II: FMD (1800 mg) and CLD (600 mg) every 12 h for 3 days (n = 54).	
The cure rates for Group I and Group II were 91.3 and 89.7% on day 28, respectively.	
A total of 36 Gabonese children 7–14 years with UFM were subjected to: FMD (30 mg/kg) +	[85]
CLD (5 mg/kg); FMD 30 mg/kg or CLD 5 mg/kg, every 12 h for 5 days.	
FMD + CLD or only CLD cured on day 28.	
A total of 105 Gabonese children aged 3–14 years with UFM received FMD (30 mg/kg) +	[86]
CLD (10 mg/kg) every 12 h for 3 days. 94% efficacy on day 28.	
A total of 51 Gabonese children 1–14 years old with UFM were treated with 3-day combination	[87]
of FMD (30 mg/kg) and CLD (10 mg/kg), respectively every 12 h.	
The cure rate on day 28 was only 62%.	
A total of 37 Mozambican children 6–36 months with UFM received 2 × day FMD (30 mg/kg)	[88]
and CLD (10 mg/kg): 45.9% cure on day 28.	
A total of 50 Gabonese children with UFM were treated with AS-FMD (1 to 2 mg/kg and 30 mg/kg,	[89]
respectively), every 12 h on 2 or 4-day regimens. A 3-day regimen or longer achieved 100% cure on day 28.	
**Ivermectin**	
In London, 25 healthy volunteers received IVM (200µg/kg) or placebo. One day later, mosquitoes were fed	[113]
on volunteers and their mean survival was 2.3 days (IVM group) and 5.5 days (control group):	
mosquito mortality was 73, 84, and 89% on days 2, 3, and 4, respectively in the IVM group.	
No differences were found between the groups when mosquitos were fed 14 days after treatment.	
In Burkina Faso, healthy patients with at least 90 cm in height received a single dose	[118]
of IVM (150–200 µg/kg) and albendazole (400 mg) (control group n = 233). The intervention group (n = 330) received	
5 more doses of IVM at 3-week intervals over the 18-week treatment phase. Incidence of	
malaria in the intervention group was 2 episodes per child and in the control group 2.39 episodes,	
showing that mass drug administration of IVM reduced malaria episodes during the transmission season.	
Controlled human malaria infection trial, in malaria naïve volunteers in Germany: 8 out 12 participants	[122]
received IVM 0.4 mg/kg once 2 h before being infected intravenously with 3200 *P. falciparum* sporozoites.	
No significant effect on parasitemia, showing that this dose of IVM has no major effect on the liver stage of *P. falciparum*.	
In Kenya, adults with UFM received 3 days of IVM at 300 (n = 48), 600 µg/kg (n = 47) or placebos (n = 46) + 3 days of DHA-PPQ. *A. gambiae* were fed with blood taken of patients on days 0.2 + 4 h, 7, 10, 14, 21, and 28 days post-treatment. Mosquito survival was checked daily until day 28 after feeding. Mosquito fed on blood taken 7 days after treatment showed the higher mortality rate of 96, 92, and 41%, to 600 µg/kg, 300 µg/kg, and placebo, respectively.	[124]

CTM: cotrimoxazole; TM: trimethoprim; SFM: sulfamethoxazole; UFM: uncomplicated falciparum malaria; SP: sulfadoxine – pyrimethamine combination; ERY: erythromycin; FMD: fosmidomycin; CLD: clindamycin; AS: artesunate; IVM: ivermectin; DHA: dihydroartemisinin; PPQ: piperaquine; ART: antiretroviral therapy; pfdhfr: *Plasmodium falciparum* dihydrofolate reductase; pfdhps: *Plasmodium falciparum* dihydropteroate synthase.

## Data Availability

Data sharing not applicable.
